# Completing a molecular timetree of primates

**DOI:** 10.3389/fbinf.2024.1495417

**Published:** 2024-12-16

**Authors:** Jack M. Craig, S. Blair Hedges, Sudhir Kumar

**Affiliations:** ^1^ Institute for Genomics and Evolutionary Medicine, Temple University, Philadelphia, PA, United States; ^2^ Department of Biology, Temple University, Philadelphia, PA, United States; ^3^ Center for Biodiversity, Temple University, Philadelphia, PA, United States

**Keywords:** primates, phylogeny, dating, evolution, speciation

## Abstract

Primates, consisting of apes, monkeys, tarsiers, and lemurs, are among the most charismatic and well-studied animals on Earth, yet there is no taxonomically complete molecular timetree for the group. Combining the latest large-scale genomic primate phylogeny of 205 recognized species with the 400-species literature consensus tree available from TimeTree.org yields a phylogeny of just 405 primates, with 50 species still missing despite having molecular sequence data in the NCBI GenBank. In this study, we assemble a timetree of 455 primates, incorporating every species for which molecular data are available. We use a synthetic approach consisting of a literature review for published timetrees, *de novo* dating of untimed trees, and assembly of timetrees from novel alignments. The resulting near-complete molecular timetree of primates allows testing of two long-standing alternate hypotheses for the origins of primate biodiversity: whether species richness arises at a constant rate, in which case older clades have more species, or whether some clades exhibit faster rates of speciation than others, in which case, these fast clades would be more species-rich. Consistent with other large-scale macroevolutionary analyses, we found that the speciation rate is similar across the primate tree of life, albeit with some variation in smaller clades.

## Introduction

**FIGURE 1:**
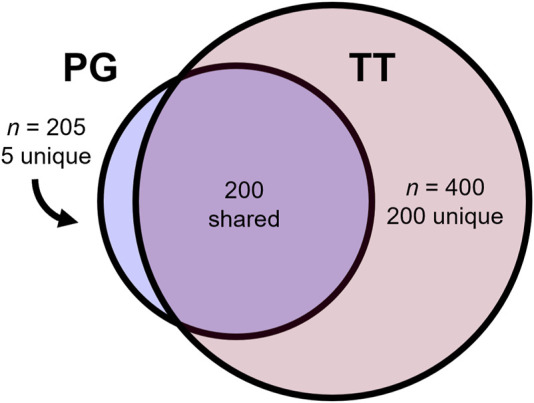
Comparison between the phylogenomic (PG) and TimeTree.org (TT) timetrees. The PG tree included 205 primates, of which five were absent from the TT, while the TT phylogeny included 400 primates, 200 of which were absent from the PG.

The mammalian order of Primates comprises 172 species of Old World apes and monkeys (*Catarrhini*), 146 New World monkeys (*Platyrrhini*), and 144 lemurs, lorises, and galagos (*Strepsirrhini*) out of a total of 462 primates in the NCBI taxonomy resource. The largest phylogenomic (PG) evolutionary tree of primates to date (Kuderna et al., 2024) required the assembly of 187 novel primate reference genomes ranging from 2.1 to 3.0 Gb in size and their alignment with 52 existing reference genomes. This produced an alignment that spanned roughly 52% of all primate species found in the NCBI taxonomy browser. An even larger molecular super-timetree of 400 primate species is available from TimeTree.org (TT) (Kumar et al., 2022), representing the synthesis of more than 4,100 published molecular timetrees across three Figure 1 decades of research, 87 of which include NCBI primate species divergences. This tree contains 200 of the same species as the PG tree, while five primate species remain unique to the PG tree. Thus, the TT and PG trees together include 405 unique NCBI species (Figure 1). This leaves 57 further species required to build a comprehensive molecular timetree of primates. Seven of these do not have molecular data in NCBI GenBank, precluding their inclusion.

We followed a three-step protocol (Craig et al., 2023a) to add the remaining 50 species into the global primate timetree: (1) a rigorous literature search for timed molecular phylogenies that contain any of the 50 missing species; (2) a subsequent search for molecular phylogenies with branch lengths that could be scaled to time for the remaining species; and (3) assembly of novel sequence alignments from data on GenBank and timetree construction for any species still missing after the first two steps. Using all three techniques, we report the assembly of a molecular phylogeny of 455 primates.

Using the resulting nearly complete phylogeny, we conducted macroevolutionary analyses. We compared the species richness of five major primate lineages to their crown age and their intrinsic rates of speciation for testing whether primate species richness accumulates at a constant rate through time (Coyne and Orr, 1998; McPeek and Brown, 2007; Hedges et al., 2015; Marin and Hedges, 2016; Henao Diaz et al., 2019), correlating with age, or whether some clades produces new species faster than others (Sanderson and Donoghue, 1994; Wilson, 2003; Fontanillas et al., 2007; Boucher et al., 2017; Sayol et al., 2019; Han et al., 2020). Thus, our new timetree represents the most complete description of the evolutionary relationships among primates to date, allowing us to map the pattern of lineage divergences through time and characterize the evolutionary forces shaping primate biodiversity.

## Results

### The expanded timetree of primates

Of the 462 primate species recognized by the NCBI taxonomy resource (Schoch et al., 2020), two monkeys (*Cheracebus medemi* and *Callicebus oenanthe*) and five lemurs (*Cheirogaleus andysabini*, *Cheirogaleus grovesi*, *Cheirogaleus minusculus*, *Cheirogaleus shethi,* and *Hapalemur gilberti*) have no molecular data annotated as a gene in NCBI GenBank. Thus, 455 primates remain, for which either published molecular phylogenies or sequences accessed to GenBank are available. These would be the target species for our supertree of all primates.

We began synthesizing these 455 primates with the nuclear genomic phylogeny found in Kuderna et al. (2024), which included 205 of our target primate species. We then acquired a primate phylogeny from TimeTree, a phylogenetic database synthesizing 4,185 published molecular phylogenies, including 148,876 species (Kumar et al., 2022). We found 200 of the remaining primate species among these, leaving a further 50. Of these, 26 missing species were found in a recently published timetree of apes and monkeys (Craig et al., 2023b).

For the remaining 24 missing primate species, we conducted a multifaceted literature search for publications containing phylogenetic trees. First, we identified the source studies for genetic samples from these species deposited in GenBank (Clark et al., 2016), assuming these may have been used to build molecular phylogenies. We also searched Google Scholar for any mention of these species in a phylogenetic context, using the same approach employed by TimeTree (Kumar et al., 2022), but with the benefit of a target species list and without limiting our search to timed phylogenies. This yielded seven published primate phylogenies containing 20 species (Springer et al., 2012; Lei et al., 2017; Masters et al., 2017; Salmona et al., 2018; Sgarlata et al., 2019; Hagemann et al., 2022; Blair et al., 2023) (Supplementary Table S1).

However, acquiring these phylogenies in a Newick format for synthesis was not trivial. While many journals endeavor for extensive data availability, this often does not extend to the final results and phylogenies printed in a research article. Of the seven new studies we identified in our search, only one (Lei et al., 2017) had their final timetree available as a standard Newick tree file in the supplementary information. We manually created Newick trees for the remaining six based on phylogeny figures. MEGA’s manual tree drawing tool (Tamura et al., 2021) was used to draw species relationships. Each branch’s length was set to the one measured using ImageJ (Schneider et al., 2012) from the published phylogeny. Finally, we visually inspected each tree and corrected any discrepancies by manually editing the resulting Newick string to ensure accurate reproduction of the published tree figure. These primary timetree files are available in the Supplementary Material.

Among these seven new trees, three (Springer et al., 2012; Lei et al., 2017; Masters et al., 2017) had been time-calibrated by their original authors, so they were used directly. For four others, we had phylogenies where the length of each branch represented the genetic distance (number of substitutions per site). This precluded adding them to our super-timetree directly, so we obtained the literature–consensus secondary calibration time for a given node in each tree from the TimeTree database following (Craig et al., 2023a; Craig et al., 2023b). Then, we constructed an unbiased uniform probability distribution between the upper and lower confidence intervals provided by TimeTree. This phylogeny was then scaled to time using the RelTime (Tamura et al., 2012; Tamura et al., 2018) approach in MEGA (Tamura et al., 2021). All the tree files and calibration schemes are available in the Supplementary Material.

Inclusion of these timetrees accounted for 451 primates of the 455 present in our target set, leaving just four for which no phylogenetic trees were found in the literature: *Lepilemur mitsinjoensis*, *Nycticebus hilleri*, *Phaner furcifer,* and *Xanthonycticebus pygmaeus*. NCBI GenBank contained sequence data from mitochondrial proteins for each of these species: NADH subunit 3 for *Lepilemur mitsinjoensis*, NADH subunit 4 for *N. hilleri*, CYTB for *P. furcifer,* and NADH subunits 4 and 5 for *X. pygmaeus*. Using NCBI smartBLAST, we identified GenBank accessions for these four proteins in ten or more additional closely related lemurs and exported alignments for each from GenPept. For each of the four resulting alignments, we trimmed any extra loci preceding or following the protein of interest so that all sequences covered the same range of amino acid positions. Then, we visually inspected the alignments in MEGA (Tamura et al., 2021), built a phylogeny from each alignment in MEGA (Tamura et al., 2021) using the JTT substitution model with little bootstraps (Sharma and Kumar, 2021) as a test of confidence at each node, and timed these trees using RelTime (Tamura et al., 2012; Tamura et al., 2018). All alignments, calibration schemes, and intermediary tree files are available in the Supplementary Material.

**FIGURE 2:**
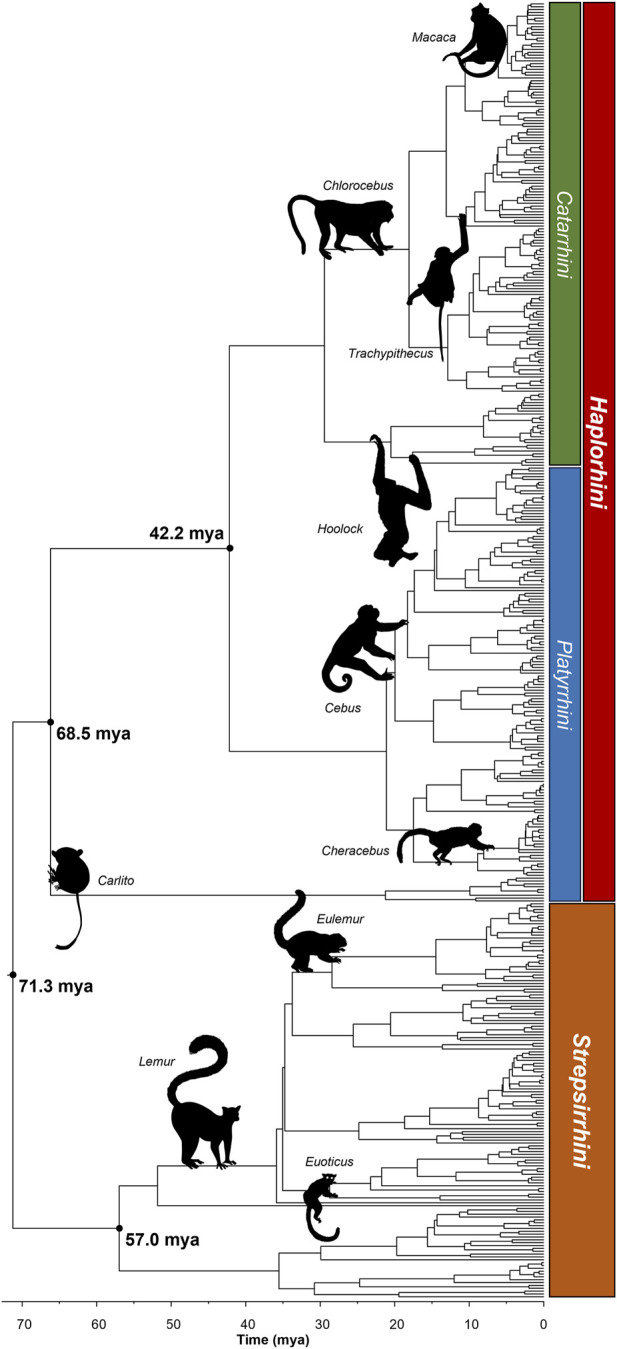
Phylogeny of 455 species of primates synthesized using Chrono-STA. The root of the phylogeny is recovered at 71.3 mya. The crown of Strepsirrhini is at 57.0 mya, and the crown of Haplorhini is at 68.5 mya. The crown of Simiiformes is at 42.2 mya. Images from Phylopic.org. The Newick tree file is available in the Supplementary Material.

Finally, we used Chrono-STA (Barba-Montoya et al., 2024) to combine all the timetrees, including the PG and TT trees, published timetrees, newly timed phylogenies, and timetrees assembled from new alignments. Chrono-STA (Barba-Montoya et al., 2024) combines timetrees based on divergence times between species. This yielded the most taxonomically complete time-calibrated primate phylogeny to date in which every tip and node age is informed by molecular data (Figure 2). It incorporates 455 primates, 98.4% of all those present in the NCBI taxonomic resource, and 100% of those with appropriate molecular data.

We recover the root of the phylogeny, covering the divergence between *Haplorhini* (apes and monkeys, 316 species) and *Strepsirrhini* (lemurs and lorises, 139 species) at 71.3 million years ago (mya). This is consistent with the time reported by TimeTree as the consensus of 48 research articles published since 1991 (71.4–77.5 mya). We estimate the crown age of *Strepsirrhini* as 57.0 mya, the crown age of *Haplorhini* as 66.2 mya, and the crown age of *Simiiformes* (apes and monkeys, or *Haplorhini* minus the tarsiers, 307 species) as 42.2 mya.

### Macroevolutionary analyses

This comprehensive molecular timetree of Primates is used to test macroevolutionary hypotheses. We use this phylogeny to compare two alternate explanations for the origin of hyper-diverse clades. First, more speciose clades may simply be older than their less diverse counterparts, allowing greater time for species to accumulate (Coyne and Orr, 1998; McPeek and Brown, 2007; Hedges et al., 2015; Henao Diaz et al., 2019). Second, they may have a faster rate of speciation due to their intrinsic characteristics, such as anatomical features tailored to their habitat, diet, or life history (Sanderson and Donoghue, 1994; Wilson, 2003; Fontanillas et al., 2007; Boucher et al., 2017; Sayol et al., 2019; Han et al., 2020). We compare these two hypotheses across the whole primate phylogeny plus four major lineages of primates: the *Strepsirrhini* and *Haplorhini*, plus the two haplorhine clades, *Platyrrhini* and *Catarrhini*. While some phylogenetic nesting is inevitable in these results, the phylogenies of each of these five clades should nonetheless be comparable for our purposes.

To test these two alternate hypotheses, we used a pair of Bayesian macroevolutionary models (Supplementary Table S2). First, the cladogenetic diversification rate shift (ClaDS) model infers rates of speciation for each lineage individually, assuming inheritance of the maternal rate with some stochasticity (*σ*) at each divergence event, which produces an overall trend in speciation (*α*) for a given clade (Maliet et al., 2019; Maliet and Morlon, 2021). For a clade evolving at a constant rate through time, we expect to see an *α* near 1.0 and a low *σ*. By contrast, the TESS model infers the trend in the rate of speciation across the entire phylogeny through time (Höhna, 2015; Höhna et al., 2016; May et al., 2016).

To test our two hypotheses about primate biodiversity, we first compared the number of species identified in our phylogeny from each clade to the crown age we recovered for that clade and identified a linear relationship (*R* ^*2*^ = 0.56), suggesting a correlation between age and species richness. Next, we extracted the mean empirical hyperparameter of the speciation rate from the TESS result for each clade and performed the same regression with species richness. However, we observed a much weaker correlation (*R* ^*2*^ = 0.10).

## Conclusion

Through the synthesis of published timed phylogenies, untimed phylogenies, and molecular sequences, we assembled a molecular phylogeny of 455 primates, excluding only seven species for which no suitable molecular data have been collected (Craig et al., 2023a). Such large-scale, taxonomically complete phylogenies are still relatively rare in the field, even for exceptionally well-studied groups (Barba-Montoya et al., 2024), but they are highly valuable for downstream work in evolutionary biology and conservation.

For example, this new tree allowed addressing some long-standing questions regarding evolution in primates, among other hyperdiverse clades of species. We observed a crown age for primates (71.3 mya) and its two major clades, *Strepsirrhini* (57.0 mya) and *Haplorhini* at (66.2 mya), which are roughly concurrent with the K-Pg boundary at 66 mya, suggesting radiation of major primate lineages following the extinction of non-avian dinosaurs. These dates are similar to those obtained in the largest genomic phylogenies (Janiak et al., 2022; Kuderna et al., 2024).

We also found that a primate clade’s crown age was a stronger predictor of its species richness than its intrinsic speciation rate. This supports the hypothesis that species richness is frequently not the product of unique adaptations driving elevated rates of speciation but instead the result of a steady accumulation of species over evolutionary time (Coyne and Orr, 1998; McPeek and Brown, 2007; Hedges et al., 2015; Marin and Hedges, 2016; Henao Diaz et al., 2019). In this model, speciation occurs primarily in isolation following the emergence of vicariant barriers to gene flow. Under these circumstances, two lineages that once represented the same species gradually accumulate genetic incompatibilities at a regular rate, establishing a molecular clock for speciation. Because vicariant barriers like rivers and mountain ranges occur randomly with respect to time and the acquisition of genetic incompatibilities is fundamentally clock-like, we can expect to observe roughly constant speciation at large enough temporal and geographical scales. Therefore, as we observe in our results, the species richness of a given clade is expected to most closely reflect its age (though small clades may experience some variations in rate due to local phenomena).

Thus, the tree we assemble here is a useful synthesis of decades of work in primate phylogenetics and, hopefully, may serve as a blueprint for future large-scale synthetic molecular trees of other well-studied groups, such as mammals.

## Methodological details

### Taxonomic reference

The TimeTree database uses the NCBI taxonomy resource (Schoch et al., 2020) for its taxonomic framework, and the same has been applied for primates. We identified 462 binomial primate taxa recognized as valid in this reference, excluding extinct species (such as *Homo heidelbergensis*), species which could not be identified (often indicated with an “*sp.*” in place of a specific name), and any hybrids, redundant subspecies, or regional variants. This list formed the basis of subsequent literature searches to identify potential timetrees for our supertree approach. Of these, *Cheracebus medemi*, *Callicebus oenanthe*, *Cheirogaleus andysabini*, *Cheirogaleus grovesi*, *Cheirogaleus minusculus*, *Cheirogaleus shethi,* and *Hapalemur gilberti* have no molecular data deposited in GenBank which had been annotated with individual genes and had appeared in no molecular phylogenies we could identify, rendering them incompatible with the synthetic tree building approach we used here. Thus, our target species list included 455 primates (98% of the 462 species included in NCBI). These 455 species represent 87% of the 525 primate species recognized by the IUCN Red List (IUCN, 2024). The additional 63 species missing from NCBI lack molecular data.

### Phylogeny processing

Phylogenies were reproduced as Newick strings from published image files for seven studies first by manually constructing the topology in the alpha release of MEGA version 12 (Tamura et al., 2021). This new feature allows users to add, remove, and reposition phylogenetic branches in a graphical user interface and then export the result as a Newick string readable by any standard phylogenetic software. Branch lengths were measured using ImageJ 1.53k (Schneider et al., 2012) by recording the length of the provided scale bar in pixels and then translating the length of each phylogenetic branch in pixels into the provided units, either millions of years for timed trees or molecular substitutions for untimed trees.

We then timed the untimed trees using a literature consensus secondary calibration approach developed in previous work (Craig et al., 2023a; Craig et al., 2023b). For each of the five untimed trees, we constructed a relative timetree using RelTime in MEGA (Tamura et al., 2012; Tamura et al., 2018; Tamura et al., 2021). We then selected a relatively basal divergence, but not the crown split, as RelTime estimated divergence times for the ingroup species and treated this as a time calibration point. We used the TimeTree database to generate a distribution of divergence times estimated in prior published work. We assumed the minimum and maximum boundaries of the confidence interval around the median estimated time as endpoints of a uniform distribution imposed on the selected node. Using this calibration, we finally converted our relative timetree into an absolute timetree.

### Phylogeny building

For the four species with molecular data existed yet no published phylogeny, we searched GenBank for a mitochondrial protein greater than 100 amino acids in length, which had been the focus of substantial prior research. This included CYTB and three subunits of NADH dehydrogenase.

We submitted these to NIH CGR SmartBLAST, and from the resulting accessions, we selected the accession with the highest percentage of shared identity for each primate species, plus the homologous human and mouse accession. We exported these to a fasta using GenPept, aligned and trimmed excess sequences from the ends where necessary, and built timetrees in MEGA (Tamura et al., 2021). We used the maximum likelihood search for each under the JTT model. We used little bootstraps (Sharma and Kumar, 2021) with an adaptive parameter search as a test of confidence in our topology. We then timed these trees using RelTime (Tamura et al., 2012; Tamura et al., 2018), providing both our alignment and the inferred ML tree, and calibrating 2–4 nodes per tree. We selected literature–consensus secondary calibrations from TimeTree as above for each genus, which was recovered as monophyletic or in the cases of genera for which fewer than half of the species were present, meaning that the deepest divergence we observed was likely not the true phylogenetic crown of the genus; we calibrated the divergence between the genus and its sister genus (the divergence between *Nycticebus* and *Loris* was calibrated this way). All alignments, calibration schemes, and intermediary tree files are available in the Supplementary Material.

### ClaDS

The cladogenetic diversification rate shift (ClaDS) model (Maliet et al., 2019; Maliet and Morlon, 2021) infers the rate of speciation for each daughter lineage of a given phylogenetic divergence based on the species richness of the descendant clade. We ran the ClaDS model in Julia 1.9.3 (Bezanson et al., 2017), using an automatic cutoff for the Bayesian process at a convergence among three concurrent Markov chains when the Gelman statistic decreased below 1.05 (Maliet and Morlon, 2021). We imposed a sampling fraction of 1.0 as we present a near-complete phylogeny.

### TESS

TESS (Höhna, 2015; Höhna et al., 2016; May et al., 2016; Fabreti and Höhna, 2022) estimates phylogeny-wide speciation through time as well as an initial hyperparameter of speciation for the whole clade. We allowed TESS to infer hyperparameters directly from each given phylogeny before each run and then ran the chain for 200,000 iterations, taking the first 10,000 as burn-in. We chose not to parameterize any mass extinctions. As in ClaDS, we imposed a sampling fraction of 1.0.

### Chrono-STA

To combine all timetrees, we ran Chrono-STA (Barba-Montoya et al., 2024) using its default parameters. We used the first release of Chrono-STA, which is publicly available from its GitHub repository: https://github.com/josebarbamontoya/chrono-sta.

## Data Availability

The datasets presented in this study can be found in online repositories. The names of the repository/repositories and accession number(s) can be found in the article/Supplementary Material.
